# False lumen rotational flow and aortic stiffness are associated with aortic growth rate in patients with chronic aortic dissection of the descending aorta: a 4D flow cardiovascular magnetic resonance study

**DOI:** 10.1186/s12968-022-00852-6

**Published:** 2022-03-28

**Authors:** Aroa Ruiz-Muñoz, Andrea Guala, Lydia Dux-Santoy, Gisela Teixidó-Turà, Maria Luz Servato, Filipa Valente, Juan Garrido-Oliver, Laura Galian-Gay, Laura Gutiérrez, Rubén Fernandez-Galera, Guillem Casas, Teresa González-Alujas, Hug Cuéllar-Calabria, Kevin M. Johnson, Oliver Wieben, Ignacio Ferreira-Gonzalez, Arturo Evangelista, Jose Rodriguez-Palomares

**Affiliations:** 1 Vall d’Hebron Institut de Recerca (VHIR), Barcelona, Spain; 2 CIBER-CV, Instituto de Salud Carlos III, Madrid, Spain; 3 Department of Cardiology, Hospital Universitari Vall d´Hebron, Barcelona, Spain; 4 Department of Medicine, Universitat Autònoma de Barcelona, Bellaterra, Spain; 5 CIBERESP, Instituto de Salud Carlos III, Madrid, Spain; 6 Department of Radiology, Hospital Universitari Vall d’Hebron, Barcelona, Spain; 7 Departments of Medical Physics & Radiology, University of Wisconsin, Madison, WI USA; 8 Instituto del Corazón. Quirónsalud-Teknon, Barcelona, Spain

**Keywords:** Aortic dissection, 4D flow CMR, Magnetic resonance imaging, Aortic stiffness

## Abstract

**Background:**

Patency of the false lumen in chronic aortic dissection (AD) is associated with aortic dilation and long-term aortic events. However, predictors of adverse outcomes in this population are limited. The aim of this study was to evaluate the relationship between aortic growth rate and false lumen flow dynamics and biomechanics in patients with chronic, patent AD.

**Methods:**

Patients with a chronic AD with patent false lumen in the descending aorta and no genetic connective tissue disorder underwent an imaging follow-up including a contrast-enhanced 4D flow cardiovascular magnetic resonance (CMR) protocol and two consecutive computed tomography angiograms (CTA) acquired at least 1 year apart. A comprehensive analysis of anatomical features (including thrombus quantification), and false lumen flow dynamics and biomechanics (pulse wave velocity) was performed.

**Results:**

Fifty-four consecutive patients with a chronic, patent false lumen in the descending aorta were included (35 surgically-treated type A AD with residual tear and 19 medically-treated type B AD). Median follow-up was 40 months. The in-plane rotational flow, pulse wave velocity and the percentage of thrombus in the false lumen were positively related to aortic growth rate (p = 0.006, 0.017, and 0.037, respectively), whereas wall shear stress showed a trend for a positive association (p = 0.060). These results were found irrespectively of the type of AD.

**Conclusions:**

In patients with chronic AD and patent false lumen of the descending aorta, rotational flow, pulse wave velocity and wall shear stress are positively related to aortic growth rate, and should be implemented in the follow-up algorithm of these patients. Further prospective studies are needed to confirm if the assessment of these parameters helps to identify patients at higher risk of adverse clinical events.

**Supplementary Information:**

The online version contains supplementary material available at 10.1186/s12968-022-00852-6.

## Background

Aortic dissection (AD) is one of the most devastating complications of the thoracic aorta and is associated with high morbidity and mortality [1]. In the recent years, outcomes in the acute phase have improved due to earlier detection, and advances in diagnostic imaging techniques and surgical and endovascular interventions [2]. Consequently, acute aortic syndromes are increasingly converting to chronic aortic diseases that require a close monitoring to avoid future complications.

Type A AD is more prevalent and requires a more aggressive management, most often replacement of the diseased part of the aorta containing the dominant entry tear, whereas for type B AD, pharmacological treatment or endovascular repair in case of complications [1] is conventionally used. Although emergent surgery for acute type A AD has significantly improved survival, most patients persist with a residual dissection of the descending aorta (surgically-treated type A with residual descending AD) [3], thereby increasing the number of patients with a chronic type B AD.

Survival rate in chronic type B AD has been established around 50–80% at 5 years and 30–60% at 10 years [4–7]. Thus, frequent clinical and imaging follow-up is recommended to evaluate the progression of aortic dilation [8] since endovascular aortic repair or open surgery is indicated in case of either fast progressive thoracic aortic enlargement (> 10 mm/year) or aortic diameter > 60 mm [8, 9]. Predictors of aortic dilation or adverse events in patients with a patent false lumen in the descending aorta after an AD have mostly been focused on morphological or anatomical variables such as the baseline aortic diameter and entry tear size and its location [10]. Additionally, partial false lumen thrombosis has been related to a higher aortic enlargement at follow-up [11, 12].

Although a patent false lumen in the descending aorta has been associated with aortic enlargement [13], need of aortic intervention/repair or late mortality [14], studies quantifying flow in the false lumen and its relationship with aortic growth rate are still limited. Studies based on 4-dimensional (4D) phase-contrast cardiovascular magnetic resonance (4D flow CMR) analysing flow dynamics in the true and false lumen have been published [15–19]. However, these studies reported qualitative or semi-quantitative analysis of flow data [16, 17], included a limited number of patients (≤ 20), and mixed individuals with and without genetic connective tissue disorders [15–19]. Additionally, the potential role of wall shear stress (WSS) and the biomechanical properties of the aortic wall (aortic stiffness) in patients with a chronic, patent false lumen in the descending aorta after an AD still remain unexplored.

This study aimed to test whether false lumen flow dynamics and biomechanics may predict aortic growth rate in a cohort of patients with a chronic, patent false lumen in the descending aorta.

## Methods

### Study population

**Fig. 1:**
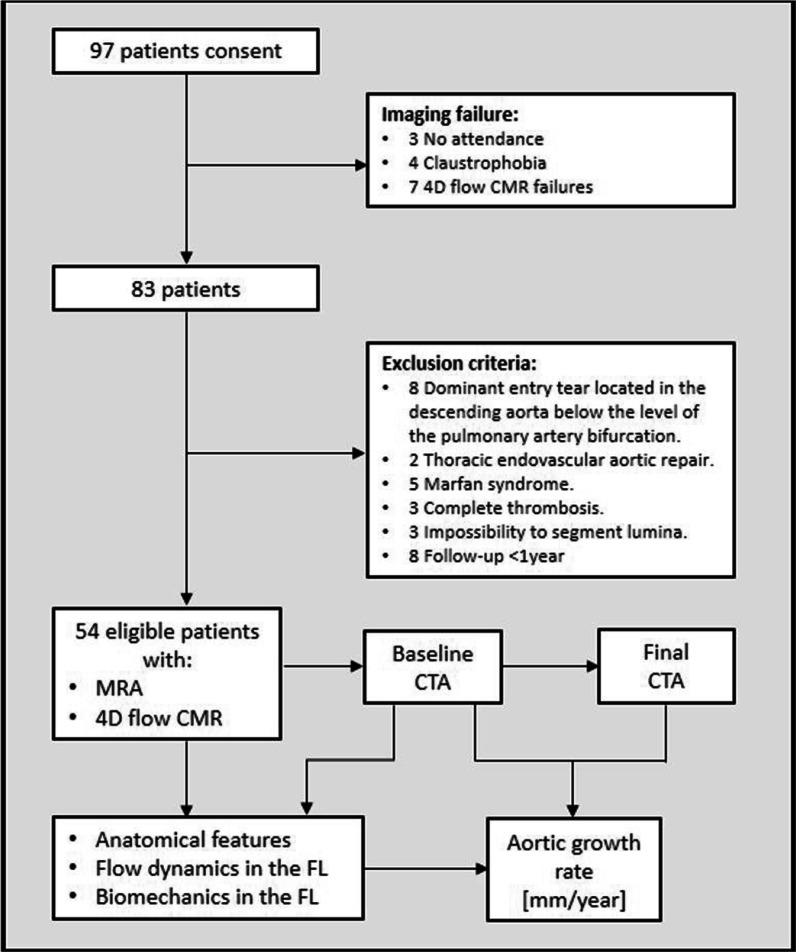
Flow chart. *CMR* cardiovascular magnetic resonance, *FL* false lumen, *MRA* magnetic resonance angiography, *CTA* Computed tomography angiography

Between November 2015 and June 2019, consecutive patients with a chronic AD and a patent false lumen in the descending aorta followed at the Aortic Unit of Vall Hebron Hospital underwent a 4D flow CMR. In January 2021, patients with at least two computed tomography angiograms (CTA) acquired at least 1 year apart and after the CMR protocol, were identified and included. Exclusion criteria were age < 18 years, bicuspid aortic valve, genetic connective tissue disorders, previous endovascular or surgical repair of the descending aorta, main entry tear located in the descending aorta below the level of the pulmonary artery bifurcation, complete false lumen thrombosis, baseline CTA study acquired < 3 months after the acute event, follow-up duration < 1 year and contraindication for CMR (Fig. 1). The study was approved by the institutional Ethics Committee and written informed consent was obtained from all participants.

### Computed tomography protocol

CTAs were acquired on a 128-slice CT scanner (Definitie AS + , Siemens, Forchheim, Germany). Arterial-phase non-electrocardiographic (ECG) gated CTA was obtained after intravenous bolus injection of 100 mL of non-ionic iodinated contrast material at a flow rate of 4 mL/s and using an automated bolus-tracking technique. Contiguous axial 1 mm sections were acquired from the thoracic inlet to the pubic symphysis.

### Cardiovascular magnetic resonance protocol

CMR studies were performed on a 1.5 T scanner (Signa, General Electric Healthcare, Waukesha, Wisconsin, USA). The protocol included a contrast-enhanced CMR angiogram to obtain the 3D volumes of the true and false lumina and two 4D flow CMR studies, one acquired with a velocity encoding (VENC) of 70 cm/s to allow accurate assessment of low velocities in the false lumen [20] and the other with a higher VENC of 150 cm/s to assess regions of high velocity flow.

A radially-undersampled acquisition (PC-VIPR [phase-contrast vastly undersampled isotropic projection]) with 5-point balanced velocity encoding [21] and retrospective-ECG gating during free-breathing was used for 4D flow CMR imaging of the entire thoracic aorta, using the following parameters: field of view 400 × 400 × 400 mm, acquisition matrix 160 × 160 × 160, voxel size 2.5 × 2.5 × 2.5 mm and flip angle 8 $^\circ$. The dataset was reconstructed according to the nominal temporal resolution of each patient, ranging from 23 to 46 ms. Data were corrected for background phase from concomitant gradients, eddy currents and trajectory errors of the 3D radial acquired k-space [21]. Brachial systolic and diastolic blood pressures were registered immediately after the CMR study.

### Aortic growth rate and anatomical features

The aortic growth rate was calculated as the difference in the diameter between the last and first available CTA divided by the time interval between them, at the level of the largest diameter on the final CTA and using the double-oblique approach. The maximum descending aortic diameter, the location and area of the dominant entry tear as well as the distance from the dominant entry tear to the surgical anastomosis in type A or to the third supraortic vessel in type B AD were defined on the baseline CTA by an experienced radiologist [22]. The dominant entry tear was defined as the largest proximal communication between the true and false lumen [23]. In patients with a patent false lumen in the descending aorta after a surgically-treated type A AD, in which a primary entry tear had been closed at surgery, the largest proximal communication between both lumina was considered to be the residual dominant entry tear.

**Fig. 2:**
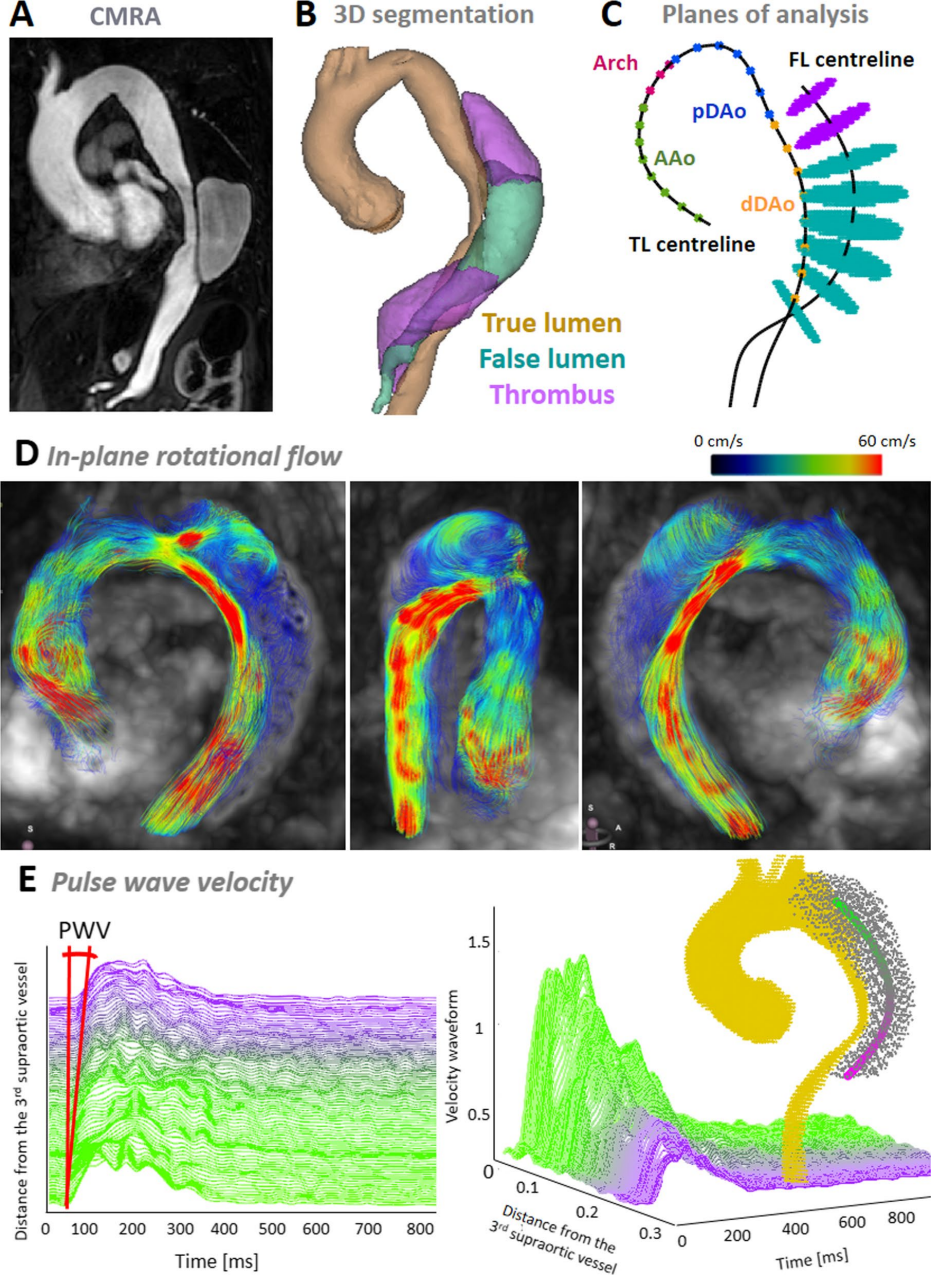
Pre-processing of imaging data: contrast-enhanced cardiovascular magnetic resonance angiography (**A**), its segmentation (**B**) and the extraction of the regions of interests in 4D flow data (**C**), and examples of rotational flow (**D**) and pulse wave velocity (**E**). *CMR* cardiovascular magnetic resonance, *CMRA* cardiovascular magnetic resonance angiography, *PWV* pulse wave velocity

The percentage of thrombus in the false lumen was calculated as the percentage of volume with thrombus with respect to the total volume of the false lumen on a contrast enhanced CMR angiography (MRA) (Fig. 2A, B).

### Flow dynamics and biomechanics in the false lumen by 4D flow CMR

Patent and thrombosed regions in the false lumen were semi-automatically segmented in the CMR angiogram (Fig. 2A, B) and six anatomical landmarks (sinotubular junction, first and third supra-aortic vessels, pulmonary artery bifurcation, diaphragmatic level, and dominant entry tear) were manually located using ITK-SNAP. These anatomical landmarks, and centreline and segmentation of both lumina were mapped to 4D flow CMR data by applying a rigid transformation. To optimize the registration, the surroundings of the transformed 3D volumes were explored with translations in the three directions after excluding the pulmonary artery, and the final location was selected as the one maximizing lumina blood flow. Then, 4D flow CMR velocity data inside the true and false lumina were extracted.

Flow dynamics in the false lumen were assessed on the low VENC 4D flow CMR study by means of a custom-designed Matlab code (Mathworks, Natick, Massachusetts, USA).

Flow rate, retrograde systolic and diastolic flow, retrograde flow fraction, in-plane rotational flow (IRF) and WSS were obtained in eight equidistant planes located perpendicular to the aortic centreline in the false lumen from the level of the pulmonary artery bifurcation to the level of the diaphragm (Fig. 2C). Only those planes at > 3 cm distal to the dominant entry tear were considered to avoid flow disturbances induced by the direct influence of the proximal entry tear. Then, for each descriptor of aortic flow dynamics the average of its values in these planes of the descending aorta was retained for the analysis.

The end of systole and the start of diastole was determined when the flow rate-time curve at the level of the sinotubular junction had reached the first 0 mL/s after the maximum peak of flow rate. Retrograde systolic and diastolic flow were calculated as the time-integral of backward through-plane flow rate curve over systolic or diastolic phases, respectively. False lumen retrograde flow fraction was calculated as the ratio of retrograde diastolic flow rate over the anterograde systolic flow rate, as proposed [18]. IRF, a parameter used to quantify flow rotation within a plane, was calculated as the integral of vorticity with respect to cross-sectional area at the systolic peak [24, 25] (Fig. 2D). Peak-systolic WSS vectors were calculated at 64 points equally-distributed along each false lumen contour by fitting the 3D velocity data and computing velocity derivatives on the vessel lumen [24, 26, 27]. Maximum systolic kinetic energy (KE), maximum systolic deceleration rate (MSDR) and flow stasis were quantified in a volume covering the false lumen from the level of the pulmonary bifurcation to the diaphragmatic level. The KE was computed as the maximum value over the cardiac cycle of the sum of the KE in all the voxels inside the volume of interest. MSDR was computed as the maximum minus the minimum systolic acceleration divided by the time interval between these two acceleration peaks [28]. Flow stasis was calculated as the percentage of cardiac time phases with a mean velocity < 5 cm/s, as suggested [29]. Details about the assessment of each flow descriptor and their inter-observer and intra-observer reproducibility are included in Additional file 1 (Table S1, Figures S2-S7).

Regional aortic pulse wave velocity (PWV) was computed from 4D flow CMR data in the false lumen as previously described [30]. Transit time was calculated with the wavelet-based method, the most robust technique [31] (Fig. 2E).

### Statistical analysis

Continuous variables are expressed as mean ± standard deviation if normally distributed and as median [1st-3rd] quartiles otherwise. Categorical variables are presented as frequency (percentage). The Kolmogorov–Smirnov test was used to assess distribution normality. Differences among groups for continuous parameters were assessed by Student’s t-test if normally distributed and Mann–Whitney U test otherwise. Chi-square test was used for categorical variables. Multivariate linear regression with a backward selection procedure was used to identify statistically-significant independent associations with aortic growth rate. Independent variables entered the model if p < 0.2 on univariate analysis and were progressively excluded if p > 0.1. The inter-observer and intra-observer reproducibility for flow descriptors were evaluated using correlation and Bland–Altman plots, Pearson correlation coefficient (R) and intra-class correlation coefficients (ICC) (average measures, two-way mixed, absolute agreement) (Supplementary Materials). A two-tailed p-value < 0.05 was considered statistically significant. SPSS (version 21.0, Statistical Package for the Social Sciences, International Business Machines, Inc., Armonk, New York, USA) was used for the analysis.

## Results

### Demographic and clinical data

From a total cohort of 97 patients with a chronic AD of the descending aorta, 54 patients met the inclusion criteria and were included in the analysis (Fig. 1). The demographic and clinical characteristics stratified by AD type are shown in Table 1. The cohort comprised 35 (65%) surgically-treated type A AD and 19 (35%) medically-treated type B AD all with a patent false lumen in the descending aorta. Mean age of the overall population was 66 ± 11 years and most patients were male (44/54, 81%). Ten (19%) patients had a mechanical prosthetic aortic valve. Twenty-four (44%) patients received anticoagulation and/or statin therapy, and 14 (26%) were treated with antiplatelet agents. Most patients had a history of hypertension (34/54, 65%) while few had a history of diabetes (5/54, 10%) or dyslipidaemia (12/54, 23%). No subject had a genetic connective tissue disorders, thus avoiding bias in aortic dilation due to genetic disease. Demographics and clinical variables were similar in type A and type B AD, however, mechanical aortic valve and antiplatelet therapy were more prevalent in surgically-treated type A than in type B AD (p = 0.058 and 0.051, respectively). The median time interval between the acute AD and the 4D flow CMR study was 51 [15; 128] months.

**Table 1:** Demographics and clinical data

Characteristics	Overall (n = 54)	Surgically-treated type A AD with residual tear (n = 35)	Medically-treated type B AD (n = 19)	P-value
Age [years]	66 ± 11	68 ± 11	64 ± 10	0.174
Sex [male]	44 (81%)	27 (77%)	17 (89%)	0.265
BSA [m^2^]	1.9 ± 0.2	1.9 ± 0.2	1.9 ± 0.2	0.701
SBP [mmHg]	138 ± 20	140 ± 21	134 ± 20	0.278
DBP [mmHg]	79 ± 13	76 ± 12	84 ± 15	0.128
Stroke volume [mL]	55 ± 19	53 ± 19	57 ± 20	0.488
Active smoker	10 (19%)	6 (18%)	4 (21%)	0.800
Hypertension	34 (65%)	21 (64%)	13 (68%)	0.727
Diabetes mellitus	5 (10%)	3 (9%)	2 (10%)	0.866
Dyslipidaemia	12 (23%)	7 (21%)	5 (26%)	0.674
Mechanical prosthetic aortic valve	10 (19%)	9 (26%)	1 (5%)	0.058
Anticoagulation therapy	24 (44%)	18 (51%)	6 (32%)	0.163
Antiplatelet therapy	14 (26%)	12 (35%)	2 (10%)	**0.051**
Statin therapy	22 (44%)	14 (45%)	8 (42%)	0.833
Heart rate [bpm]	63 ± 10	65 ± 10	61 ± 9	0.330
Follow-up duration between baseline and final CTAs [months]	35 ± 11	33 ± 11	34 ± 12	0.878
Time between acute AD and 4D flow [months]	78 ± 81	95 ± 92	47 ± 43	0.099
Aortic growth rate [mm/year]	1.8 ± 1.6	1.7 ± 1.6	1.9 ± 1.6	0.550

Values are mean ± standard deviation or n (%)

*AD* aortic dissection, *BSA* body surface area, *CTA* computed tomography angiogram, *SBP and DBP* systolic and diastolic blood pressure, respectively

Over a median follow-up duration of 40 months, interquartile range (IQR) [24; 44] months, the median aortic growth rate was 1.2 [0.6; 2.7] mm/year.

### Predictors of aortic growth rate

Anatomical features, flow dynamics and biomechanics in the false lumen stratified by AD type are shown in Table 2. Bivariate and multivariate relationships between the investigated descriptors and aortic growth rate are reported in Table 3.

**Table 2:** Anatomical features, flow dynamics and biomechanics in the false lumen

	Overall (n = 54)	Surgically-treated type A AD with residual tear (n = 35)	Medically-treated type B AD (n = 19)	P-value
*Anatomical features*				
Maximum DAo diameter [mm]	48 ± 10	48 ± 11	49 ± 8	0.368
Dominant entry tear area [cm^2^]	1.2 ± 0.8	1.1 ± 0.8	1.3 ± 0.9	0.326
Local distance of the dominant entry tear [mm]	28 ± 28	30 ± 26	26 ± 33	0.370
Thrombus in the false lumen [%]	11 ± 14	11 ± 14	12 ± 16	0.636
*Flow dynamics in the false lumen*				
Retrograde systolic flow [mL]	1.6 ± 1.3	1.6 ± 1.1	1.6 ± 1.5	0.867
Retrograde diastolic flow [mL]	9.7 ± 5.0	9.8 ± 4.5	9.5 ± 6.0	0.553
Retrograde flow fraction [%]	82 ± 42	92 ± 42	66 ± 38	0.109
IRF [cm^2^/s]	1.25 ± 5.79	1.04 ± 4.55	1.63 ± 7.72	0.923
WSS [N/m^2^]	0.26 ± 0.10	0.26 ± 0.10	0.24 ± 0.10	0.475
KE [mJ]	0.62 ± 0.48	0.54 ± 0.34	0.47 ± 0.30	0.523
MSDR [cm/s^3^]	2996 ± 2552	3448 ± 2531	2193 ± 2453	0.055
Flow stasis [%]	52 ± 18	54 ± 18	47 ± 18	0.200
*Biomechanics in the false lumen*				
PWV [m/s]	7.7 ± 3.7	7.7 ± 3.7	7.6 ± 3.6	0.808

Values are mean ± standard deviation

*DAo* descending aorta, *IRF* in-plane rotational flow, *KE* kinetic energy, *MSDR* maximum systolic deceleration rate, *PWV* pulse wave velocity, *WSS* wall shear stress

**Table 3:** Bivariate and multivariate correlation of demographics, anatomical features, and flow dynamics and biomechanics in the false lumen with aortic growth rate

	Bivariate		Multivariate		
	R	P-value	Beta	P-value	95% CI
*Demographics and clinical data*					
Age [years]	0.018	0.899			
Sex (male)		0.398			
Body surface area	0.101	0.494			
Systolic blood pressure [mmHg]	0.086	0.566			
Diastolic blood pressure [mmHg]	0.071	0.637			
Smoking		0.390			
Hypertension		0.386			
Diabetes mellitus		0.391			
Dyslipidaemia		0.396			
Mechanical prosthetic aortic valve		0.397			
Anticoagulation therapy		0.396			
Antiplatelet therapy		0.394			
Statin therapy		0.394			
Type of aortic dissection		0.481			
*Anatomical features*					
Maximum DAo diameter [mm]	0.280	**0.042**			
Dominant entry tear area [cm^2^]	0.257	0.096	0.371	**0.018**	[0.130; 1.251]
Local distance of the dominant entry tear [mm]	-0.094	0.513			
Thrombus in the false lumen [%]	0.336	**0.037**	0.293	**0.049**	[0.000; 0.065]
*Flow dynamics in the false lumen*					
Retrograde systolic flow [mL]	0.165	0.237			
Retrograde diastolic flow [mL]	0.102	0.465			
Retrograde flow fraction [%]	0.060	0.708			
IRF [cm^2^/s]	0.393	**0.006**	0.433	**0.008**	[0.034; 0.202]
WSS [N/m^2^]	0.279	0.060			
KE [mJ]	0.015	0.919			
MSDR [cm/s^3^]	-0.214	0.136			
Flow stasis [%]	0.106	0.457			
*Biomechanics in the false lumen*					
PWV [m/s]	0.370	**0.017**	0.360	**0.017**	[0.031; 0.281]

*DAo* descending aorta, *IRF* in-plane rotational flow, *KE* kinetic energy, *MSDR* maximum systolic deceleration rate, *PWV* pulse wave velocity, *WSS* wall shear stress

#### Clinical and anatomical features

Demographic (age, sex and body surface area (BSA)) and clinical characteristics (blood pressure and treatment with anticoagulants, antiplatelet agents and/or statins), comorbidities (hypertension, diabetes mellitus and dyslipidaemia), the presence of a mechanical prosthetic aortic valve, and the type of AD were not related to aortic growth rate in bivariate analysis (Table 3).

**Fig. 3:**
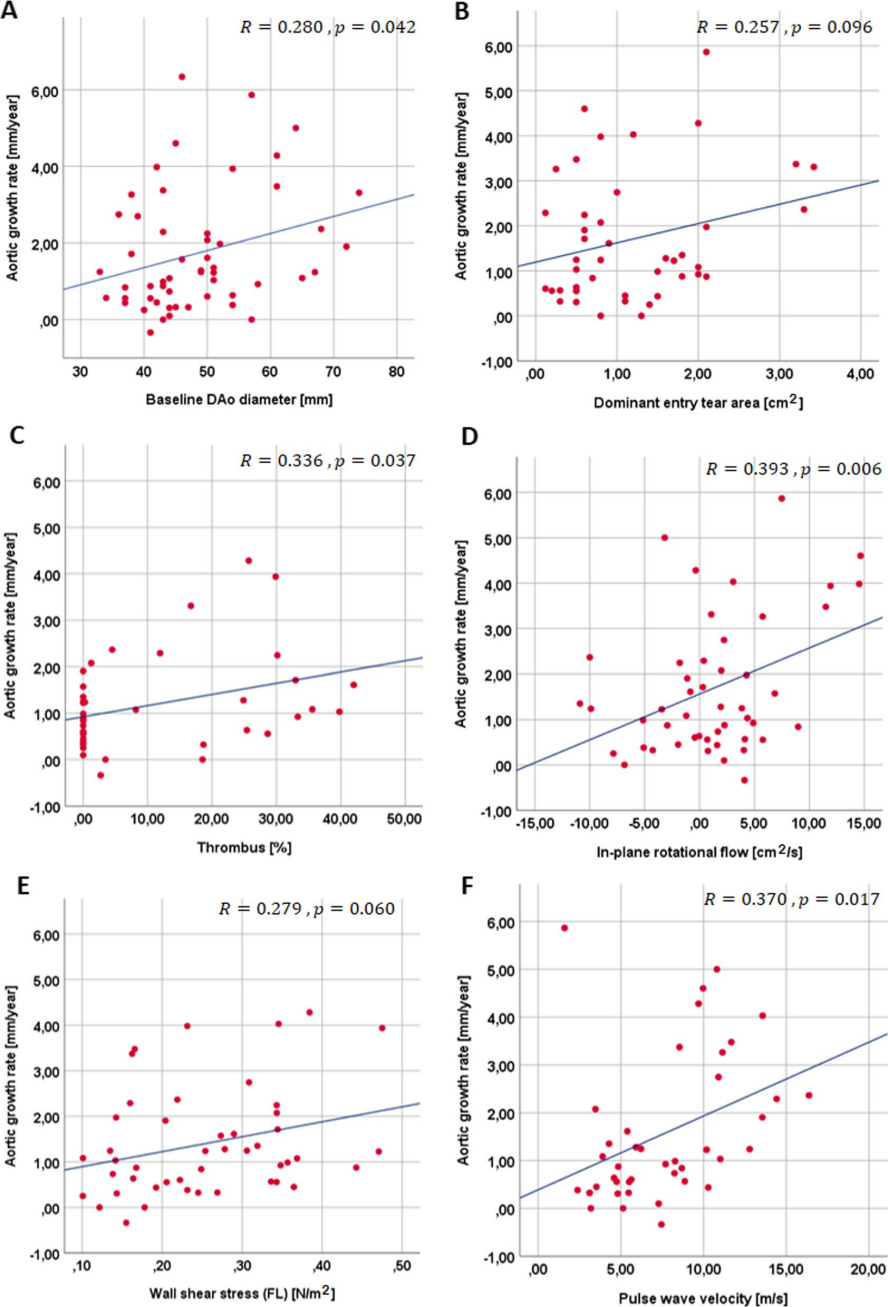
Scatter plots showing correlations between aortic growth rate and maximum diameter of the descending aorta at baseline (**A**), dominant entry tear area (**B**), thrombus (**C**), in-plane rotational flow (**D**) wall shear stress (**E**), and pulse wave velocity (**F**) in the false lumen. *AD* aortic dissection, *FL* false lumen

The median of the maximum descending aortic diameter at baseline was 45 [40; 54] and 46 [43; 54] mm in type A and B AD, respectively (Table 2). In the whole cohort it was significantly and positively related to aortic growth rate (R = 0.280, p = 0.042) (Fig. 3A). Median dominant entry tear area was non-significantly higher in type A compared to type B AD (1.0 [0.5; 1.6] vs. 0.8 [0.6; 1.9], respectively). In the whole population, it was 0.9 cm^2^, ranging from 0.12 to 10 cm^2^ (Table 2), and was positively but not significantly associated with aortic growth rate (R = 0.257, p = 0.096) (Fig. 3B). The location of the dominant entry tear was similar in both types of AD. Thrombus was present in 49% of the population, occupying between 1 and 42% of the false lumen volume, it was similar in type A and type B AD and was positively related to aortic enlargement rate (R = 0.336, p = 0.037) (Fig. 3C).

#### Flow dynamics and biomechanics in the false lumen

Flow dynamics was not distinct in type A and type B AD, only MSDR was increased in type A compared to type B AD (3123 [1081; 4999] vs. 1576 [950; 2722]) (Table 2). Systolic and diastolic retrograde flow, retrograde flow fraction, KE, MSDR and flow stasis in the false lumen were not related to aortic growth rate (Table 3). Median WSS was 0.25 [0.18; 0.34] and 0.23 [0.15; 0.34] N/m^2^ in type A and type B AD, respectively, and 0.25 [0.16; 0.34] N/m^2^ in the whole population. It showed a positive, nearly statistically-significant association with aortic growth rate (R = 0.279, p = 0.060) (Fig. 3E). Median IRF was non-significantly lower in type A than in type B AD (1.60 [− 1.80; 4.09] vs. 0.76 [− 4.30; 7.17]). It was 1.3 [− 1.9; 4.2] cm^2^/s in the whole population and was positively related to aortic enlargement rate (R = 0.393, p = 0.006) (Fig. 3D). Thus, a larger rotational component of blood flow and an increased stress conveyed by blood flow to the aortic wall were associated with faster growth.

Regarding local stiffness, median PWV was 6.8 [4.5; 10.9] m/s in type A AD, 7.7 [4.8; 10.0] m/s in type B AD and 7.4 [4.8; 10.5] m/s in the entire cohort. It was positively related to growth rate (R = 0.370, p = 0.017) (Fig. 3F). Therefore, a stiffer false lumen was associated with faster local growth.

#### Multivariate analysis

In a multivariate model corrected for dominant entry tear area and the percentage of false lumen occupied by thrombus, both IRF and PWV were independently and positively related to aortic growth rate (p = 0.008 and p = 0.017, respectively) (Table 3).

### Impact of aortic dissection type

As prognosis for surgically-treated type A AD or medically treated type B AD is different; anatomical features, and false lumen flow dynamics and biomechanics were analyzed, separately, according to the type of AD (Table 2). No differences were found between both types of AD in terms of growth rate, dominant entry tear area, thrombus in the false lumen, IRF, WSS and PWV. Moreover, the inclusion of the type of AD into the multivariate model reported in Table 3 did not provide any added predictive value for growth rate (p = 0.864).

### Reproducibility of flow descriptors

Details of inter-observer and intra-observer variability for flow descriptors are shown in Additional file 1 (Table S1, Figures S2-S7). Inter-observer and intra-observer reproducibilities were good for maximum velocity, retrograde systolic flow, retrograde flow fraction, IRF, WSS and KE (ICC between 0.75 and 0.90) and excellent for retrograde diastolic flow and flow stasis (ICC > 0.90). MSDR showed good inter-observer and excellent intra-observer agreement.

## Discussion

This study reported a comprehensive analysis of the relationship between anatomical features, flow dynamics and biomechanical descriptors in the false lumen with aortic growth rate in a cohort of patients with a patent false lumen in the descending aorta after a surgically-treated type A or medically-treated type B AD. The main findings of this work were that in-plane rotational flow, false lumen stiffness and the proportion of false lumen occupied by thrombus were positively and independently related to aortic growth rate. Additionally, wall shear stress showed a positively nearly-significant association with aortic growth rate. This is the largest 4D flow CMR study aiming at the assessment of predictors of progressive dilation for chronic descending AD.

### Anatomical features

Imaging studies suggested that complete thrombosis of the false lumen has beneficial prognostic value [32], whereas a patent false lumen predicts poor outcome and progressive aortic dilation [6, 10, 11, 13, 33]. On the other hand, there are discrepancies with respect to the role of partial thrombosis for aortic adverse events and growth rate in type B AD. In most previous studies, the existence and distribution of thrombus was determined semi-quantitatively. This is the first study to perform a volumetric quantification of the presence of thrombus in the false lumen and to evaluate its role in aortic dilation. Our study is in line with a previous study which demonstrated that new or increased false lumen thrombosis area during follow-up was strongly associated with a faster aortic growth and late adverse events [12].

### Flow dynamics and biomechanics in the false lumen

The potential role of rotational flow in promoting aortic enlargement has been suggested in previous studies based on other aortic conditions such as in the presence of bicuspid aortic valve and Marfan syndrome [24, 27, 34]. To the best of our knowledge, this is also the first quantitative study assessing rotational flow patterns and WSS in the false lumen of chronic AD of the descending aorta. Qualitatively-assessed, marked systolic helical flow in the false lumen was reported [16] and was related to the rate of aortic growth rate in bivariate analysis [17]. These preliminary qualitative results were here confirmed with quantitative data, since IRF was independently associated with aortic growth. Present results further specify the independent predictive value of rotational flow on top of established predictors, such as entry tear area [10], whose role was confirmed in the present cohort. We speculate that a large rotational flow may result in increased stress to the aortic wall that would induce a greater aortic growth (similarly to other aortic diseases). Additionally, it may also indicate a limited flow progression within the false lumen, possibly associated with higher local pressure. Therefore, these two factors (higher pressure and increased wall shear stress) could jointly induce aortic dilation. This hypothesis should be demonstrated in larger scaled studies.

Although wall shear stress has been shown to affect aortic dilation in bicuspid aortic valve and Marfan syndrome patients [24, 27, 34, 35], no previous study investigated its potential role in chronic descending AD. A pathological wall shear stress in the false lumen may play a similar role to that found in the other pathologies confined to the ascending aorta [35, 36]. In this study a positive, nearly-statistical significant association was found between WSS and aortic growth rate (p = 0.060), but this relationship was not sustained in multivariate analysis. However, the in-plane rotational flow, which is a measurement of rotational flow similar to circumferential WSS [34], was found to be independently and positively related to aortic growth rate (p = 0.008). As commented above, we hypothesized that the presence of rotational flow in the false lumen may be related to the difficulty of the flow to move forward the false lumen and the limited drainage at the exit tear/s, which may lead to higher pressurization in the false lumen and thus may contribute to aortic dilation. Further prospective studies including a larger number of patients are needed to confirm these hypothesis.

The relationship of KE and flow stasis in the false lumen with aortic growth rate had not been previously analysed in patients with chronic descending AD. This study reported that KE, flow stasis and MSDR were not associated with aortic growth rate. Our results concur with those of Marlevi et al. who found the MSDR to be equal between stable and enlarging growth groups [37]. We speculate that a larger cohort may be needed to unveil the possible role of KE and flow stasis in the prediction of growth rate in descending AD.

Confirming findings in other conditions [38, 39], this study showed that PWV is an excellent predictor for aortic dilation. In chronic descending AD, an increased PWV, indicating an increased stiffness of the false lumen wall, could furthermore induce resistance in flow progression. Indeed, for a given volume of blood filling the false lumen, higher stiffness would result in higher false lumen pressure. This finding would be in line with the observations of Burris et al. [37] in which a higher false lumen ejection fraction (associated with lack of flow progression due to greater false lumen pressure) is associated with a greater aortic dilation.

Although long-term outcomes of patients with surgically-treated type A and medically-treated type B AD are distinct [10], the increased helicity of flow and aortic stiffness are conditions observed independently of the type of AD for predicting faster growth rate. This confirms the role and strength of the predictors determined in the present study.

### Limitations

Despite being the largest study to date testing aortic flow dynamics and stiffness as predictors of aortic dilation in chronic non-complicated descending AD, the size of the cohort is still only modest. This was partially due to the exclusion of patients with genetic connective tissue disorders, whose evolution may substantially differ. Another limitation was the variability of the time interval between the acute event and the 4D flow CMR study. However, all CMR studies have been performed in the chronic phase to ensure the stability of flow variables that may be more variable in the acute or subacute phase. Larger prospective studies initiated systematically after the acute event are needed to confirm present findings. Finally, the use of time-resolved segmentations of the false lumen may entail a more accurate wall shear stress quantification by addressing the effect of intimal flap motion, which is nonetheless limited in chronic dissections [8].

## Conclusions

Increased rotational flow, aortic stiffness and wall shear stress are related to aortic growth rate in patients with an uncomplicated chronic descending AD with no genetic connective tissue disorders. Clinical follow-up of these variables may help to identify patients at major risk of late adverse events or in need of further interventions or more frequent monitoring.

## Supplementary Information

**Additional file 1:** Supplementary Material: detailed description of aortic flow dynamics descriptors and inter- and intra-observer reproducibility.

## Data Availability

The datasets used and/or analysed during the current study are available from the corresponding author on reasonable request.

## Abbreviations

AD: Aortic dissection
BSA: Body surface area
CMR: Cardiovascular magnetic resonance
CMRA: Cardiovascular magnetic resonance angiography
CTA: Computed tomography angiogram
DAo: Descending aorta
ECG: Electrocardiogram
FL: False lumen
IRF: In-plane rotational flow
KE: Maximum systolic kinetic energy
MSDR: Maximum systolic deceleration rate
PWV: Pulse wave velocity
VENC: Velocity encoding
WSS: Wall shear stress
