# Supplementary material for: False lumen rotational flow and aortic stiffness are associated with aortic growth rate in patients with chronic aortic dissection of the descending aorta: a 4D flow cardiovascular magnetic resonance study
Source: J Cardiovasc Magn Reson. 2022 Mar 28;24:20. doi: 10.1186/s12968-022-00852-6 (PMC8962590; doi:10.1186/s12968-022-00852-6)
Supplement: Supplementary file 1 — Additional file 1: Supplementary Material: detailed description of aortic flow dynamics descriptors and inter- and intra-observer reproducibility. [file 12968_2022_852_MOESM1_ESM.docx]

**Additional Material**

**1. Descriptors of aortic flow dynamics in the false lumen.**

False lumen flow dynamics was characterized, either at 8 equidistant planes or in a volume of analysis, in the false lumen, from the level of the pulmonary bifurcation to the diaphragm. Retrograde systolic and diastolic flow, retrograde flow fraction, wall shear stress and in-plane rotational flow were calculated in the 8 planes of analysis located perpendicular to the centreline of the false lumen, and then the average of each descriptor in the 8 planes was used for the analysis. Conversely, maximum systolic kinetic energy, maximum systolic deceleration rate and flow stasis were quantified in the volume of analysis.

***Retrograde systolic and diastolic flow and retrograde flow fraction***

Flow volumes were calculated as the time-integral of anterograde and retrograde through-plane flow rate curves over systolic (retrograde systolic flow) and diastolic (retrograde diastolic flow) phases. Then, retrograde flow fraction was calculated as follows:

$$Retrograde flow fraction= \frac{\int_{T_{s}}^{T_{d}} V_{RDF} \left( t \right) dt}{\int_{0}^{T_{s}} V_{ASF} \left( t \right)dt}$$

where $V_{RDF}$ and $V_{ASF}$represent the retrograde diastolic flow rate and the anterograde systolic flow rate, respectively, and $T_{d}$ and $T_{s}$ are diastolic and systolic time intervals, respectively.

***Wall shear stress***

For each plane, peak-systolic WSS vectors (averaged from 1-time frame before and 2-time frames after peak) were calculated at 64 points equally distributed along each false lumen contour by fitting the 3D velocity data with B-spline surfaces and computing velocity derivatives on the vessel lumen (1,2). Contour-averaged magnitude WSS were calculated for each plane and averaged over the 8 planes of analysis.

***In-plane rotational flow***

In-plane rotational flow was quantified as the through-plane component of circulation $\left( ⎾ \right)$. To this aim, vorticity $(\omega)$ was computed in each double-oblique analysis plane and circulation $\left( ⎾ \right)$ was obtained as the integral of vorticity with respect to cross-sectional area $(S)$,$⎾=\iint\omega dS$.

***Maximum systolic kinetic energy***

The kinetic energy (KE) was quantified in each time phase as:

$$Kinetic energy \left( t \right)= \sum_{i=1}^{n} \frac{1}{2}\cdot\rho\cdot V\cdot\left| \vec{v_{i}}(t) \right|^{2}$$

where $\rho$ is the blood density assumed as 1.06 g/cm^3^, $V$ is the voxel volume, n is the number of voxels in the volume and $\left| \vec{v_{i}}(t) \right|$ is the magnitude velocity in a voxel $(i)$ at a time $(t)$. The maximum value of KE was considered in the analysis (Figure S1, B)

***Maximum systolic deceleration rate***

Flow acceleration was calculated in each voxel inside the volume of interest in the false lumen over the cardiac cycle as follows:

$$\vec{a}= \overline{\frac{v_{i}(t+1)- v_{i}(t)}{\Delta t}}$$

where $v$ is the through-plane plane velocity (Figure S1, C) and *t* the time.

The acceleration-time curve was obtained by averaging acceleration values in all the voxels for each time phase. Then, MSDR was computed as the maximum minus the minimum systolic acceleration divided by the time interval between these two acceleration peaks (Figure S1, D)(3).

***Flow stasis***

Flow stasis was calculated as the percentage of cardiac phases with a mean velocity <5 cm/s in each voxel inside the volume of analysis, and then the average of all the values in the voxels included in the volume of interest was used (4). This threshold had been defined taking into account expected noise levels of 4D flow velocity data based on the velocity encoding range and SNR-estimates (4).

**2. Descriptors of biomechanics in the false lumen: pulse wave velocity**

The false lumen pulse wave velocity was quantified in the descending aorta, which was here defined as between the third supraortic vessel to the level of the diaphragm. Velocity waveforms were extracted at equally–distributed analysis planes and transit time was calculated with the wavelet-based method (5,6). This method has demonstrated to be the most robust, especially for low temporal resolution acquisitions (5). The method consists of applying wavelet analysis to the upslope part of each velocity waveform, obtaining a spectral discretization of the main components. Comparing two waveforms pertaining to different locations provides a distribution of the time lag of each frequency component, here limited to those with frequencies <10Hz. This spectral distribution is later averaged considering as weight the magnitude of each frequency component to obtain a unique value for transit time between the two waveforms. Starting from beginning of the analyzed region, each waveform was compared to the adjacent one. The slope of the best fit line relating cumulative distance and cumulative sum of travelling times (i.e. PWV) was extracted.

**3. Inter- and intra-observer variability of flow descriptors**

Intra-observer and inter-observer variability of the different flow descriptors was assessed in a subset of 20 randomly selected patients (10 patients for each analysis), comparing the different measurements in each plane or sub volume of the descending aorta. The inter-observer and intra-observer reproducibility for flow descriptors were evaluated using correlation and Bland-Altman plots, Pearson correlation coefficient (R) and intra-class correlation coefficients (ICC) (average measures, two-way mixed, absolute agreement).

The inter-observer reproducibility was assessed with flow data measured by two different observers (Observer 1 and Observer 2) in 10 patients. To this aim, each observer segmented the aorta and located the anatomical landmarks as described in Methods. The observers were blinded to each other’s’ results. For the intra-observer variability, Observer 1 segmented the aorta and located the anatomical landmarks twice in 10 patients. A minimum of 12 months elapsed between the two sets of measurements of the observer, who was blinded to previous results.

Inter-observer variability for the flow parameters in the false lumen is shown in Figure S2, S3 and S4. Inter-observer variability was good for retrograde systolic flow, retrograde flow fraction, IRF, WSS and KE (ICC >0.75 and <0.90) and excellent for retrograde diastolic flow and flow stasis (ICC>=0.90) (Table S1). Pearson correlation coefficients between data of Observer 1 and data of Observer 2 were >0.70 for all flow descriptors (Figures S2-S4).

Intra-observer variability is presented in Figures S5, S6 and S7. Most of the flow descriptors showed an ICC>0.75 and correlation coefficients >0.75 for the two sets of data of the Observer 1. Larger mean differences and limits of agreement for retrograde flows and IRF were found in the inter-observer variability analysis compared to the intra-observer variability analysis (Table S1).

**TABLES**

| **Table S1. Inter- and intra-observer reproducibility of flow dynamic parameters.** | | | | |
| --- | --- | --- | --- | --- |
|  | **Inter-observer variability** | | **Intra-observer variability** | |
|  | Mean difference (LOA) | ICC | Mean difference (LOA) | ICC |
| Maximum velocity [cm/s] | -5.89 (-31.59, 19.81) | 0.756 | -4.60 (-24.82, 15.62) | 0.785 |
| Retrograde systolic flow [mL] | -0.40 (-2.02, 1.21) | 0.757 | -0.09 (-1.14, 0.96) | 0.715 |
| Retrograde diastolic flow [mL] | -0.40 (-4.44, 3.63) | 0.904 | 0.07 (-3.71, 3.85) | 0.900 |
| Retrograde flow fraction [%] | -2.17 (-34.07, 29.72) | 0.877 | 9.45 (-29.96, 48.85) | 0.843 |
| IRF [cm^2^/s] | -1.97 (-18.41, 14.47) | 0.777 | -0.80 (-11.79, 10.20) | 0.788 |
| WSS [N/m^2^] | -0.02 (-0.18, 0.13) | 0.751 | -0.01 (-0.18, 0.17) | 0.855 |
| KE [mJ] | -0.04 (-0.53, 0.45) | 0.884 | -0.09 (-0.67, 0.50) | 0.859 |
| MSDR [cm/s^3^] | 288 (-1490, 2068) | 0.879 | -285 (-2334, 1763) | 0.953 |
| Flow stasis [%] | -0.10 (-11.12, 10.93) | 0.954 | -0.76 (-5.40, 3.87) | 0.984 |
| Results of the comparison of flow descriptors measured by Observer 1 and Observer 2 (inter-variability) and the comparison of two sets of flow descriptors calculated by the same Observer 1 (intra-variability). Mean difference, 95% LOA and ICC are shown.  *ICC= intra-class correlation coefficient, IRF = in-plane rotational flow, LOA= limits of agreement, KE = kinetic energy, MSDR = maximum systolic deceleration rate, WSS = wall shear stress.* | | | | |

**FIGURES**

| 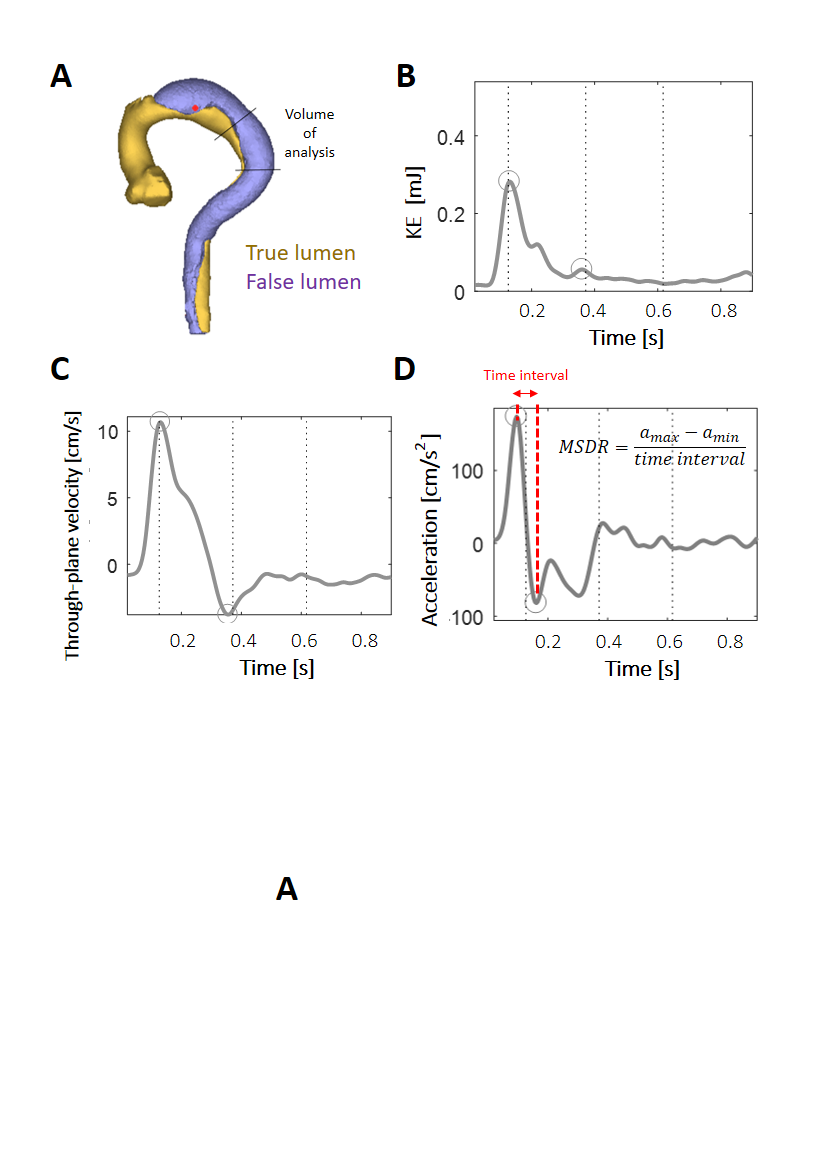 |
| --- |
| Figure S1: Kinetic energy (B), through-plane velocity (C) and acceleration-time curve (D) in a volume covering the false lumen from the pulmonary bifurcation to the diaphragmatic level (A). |

| **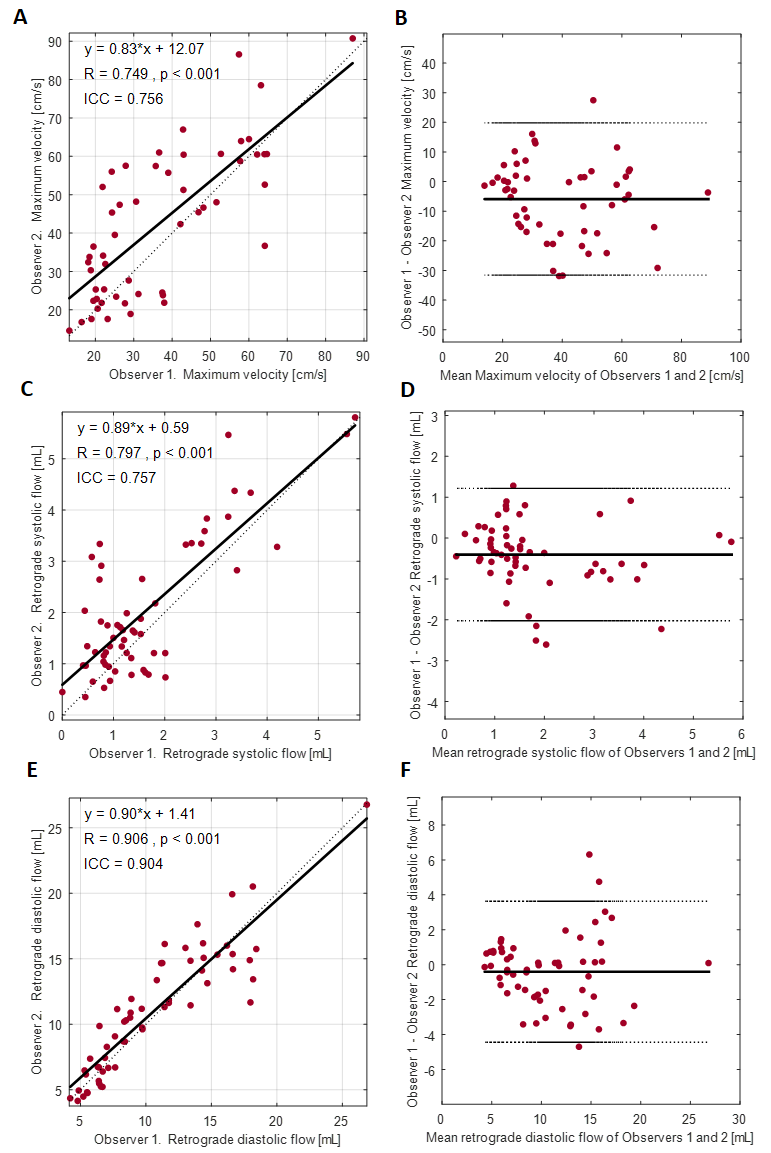** |
| --- |
| Figure S2. **I*nter-observer variability of maximum velocity and systolic and diastolic flows.*** *Correlation (left) and Bland-Altman (right) plots for the inter-observer reproducibility of maximum velocity (A and B), retrograde systolic flow (C and D) and retrograde diastolic flow (E and F). Dotted lines represent bisectors (left) and 95% confidence interval (right) while continuous lines represent best linear fit (left) and mean difference (right).* |
| **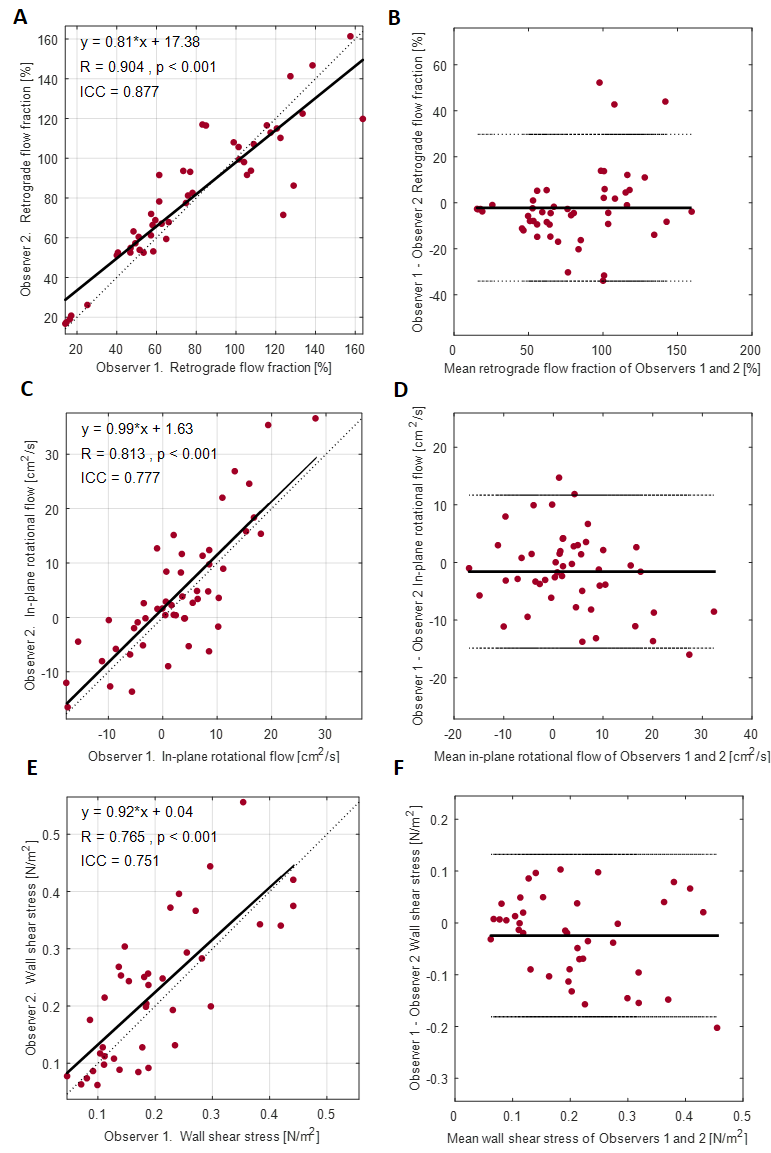** |
| Figure S3. **I*nter-observer variability of retrograde flow fraction, in-plane rotational flow and wall shear stress.*** *Correlation (left) and Bland-Altman (right) plots for the inter-observer reproducibility of retrograde flow fraction (A and B), in-plane rotational flow (C and D) and wall shear stress (E and F). Dotted lines represent bisectors (left) and 95% confidence interval (right) while continuous lines represent best linear fit (left) and mean difference (right).* |
| **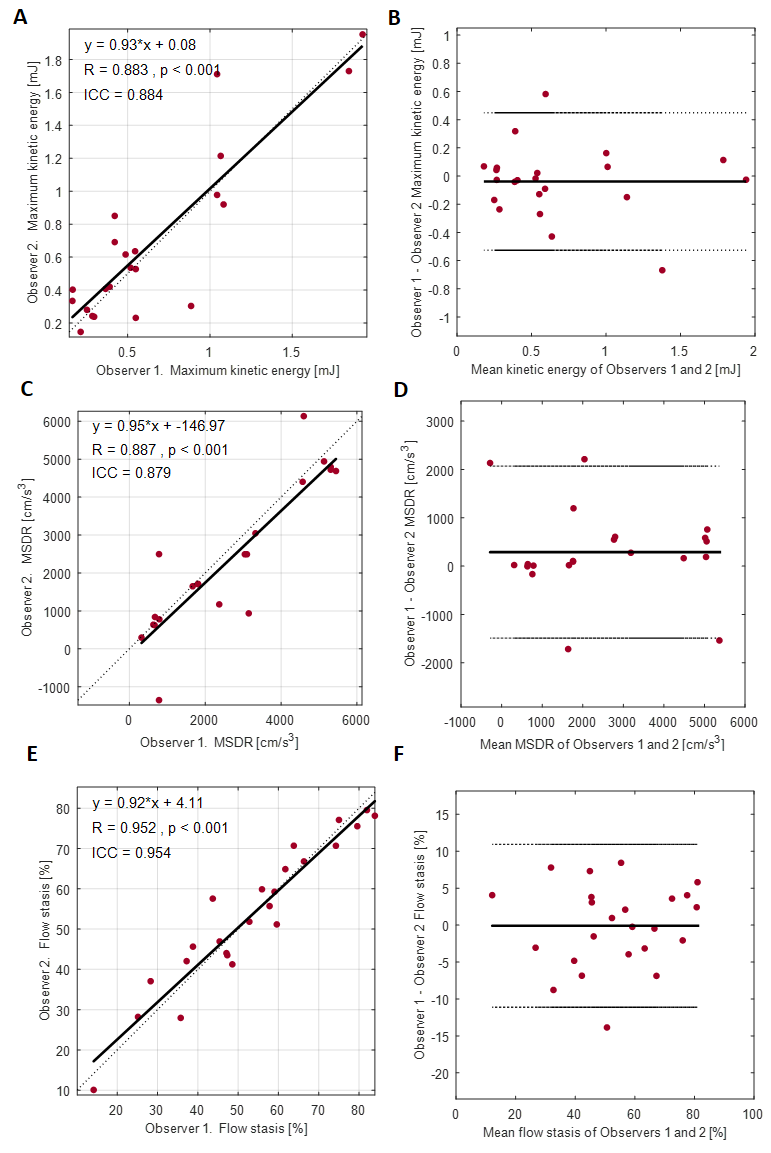** |
| Figure S4. **I*nter-observer variability of kinetic energy, maximum systolic deceleration rate (MSDR) and flow stasis.*** *Correlation (left) and Bland-Altman (right) plots for the inter-observer reproducibility of kinetic energy (A and B), MSDR (C and D) and flow stasis (E and F). Dotted lines represent bisectors (left) and 95% confidence interval (right) while continuous lines represent best linear fit (left) and mean difference (right).* |
| **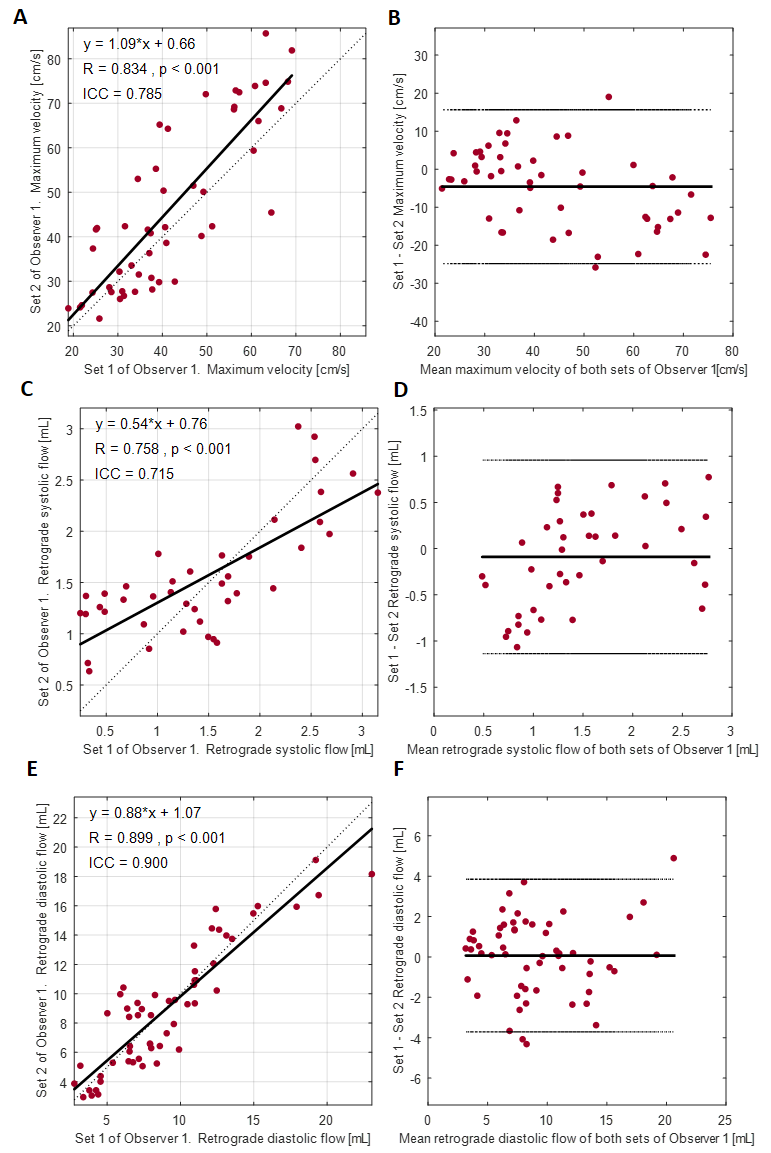** |
| Figure S5. **I*ntra-observer variability of maximum velocity and systolic and diastolic flows.*** *Correlation (left) and Bland-Altman (right) plots for the intra-observer reproducibility of maximum velocity (A and B), retrograde systolic flow (C and D) and retrograde diastolic flow (E and F). Dotted lines represent bisectors (left) and 95% confidence interval (right) while continuous lines represent best linear fit (left) and mean difference (right).* |
| **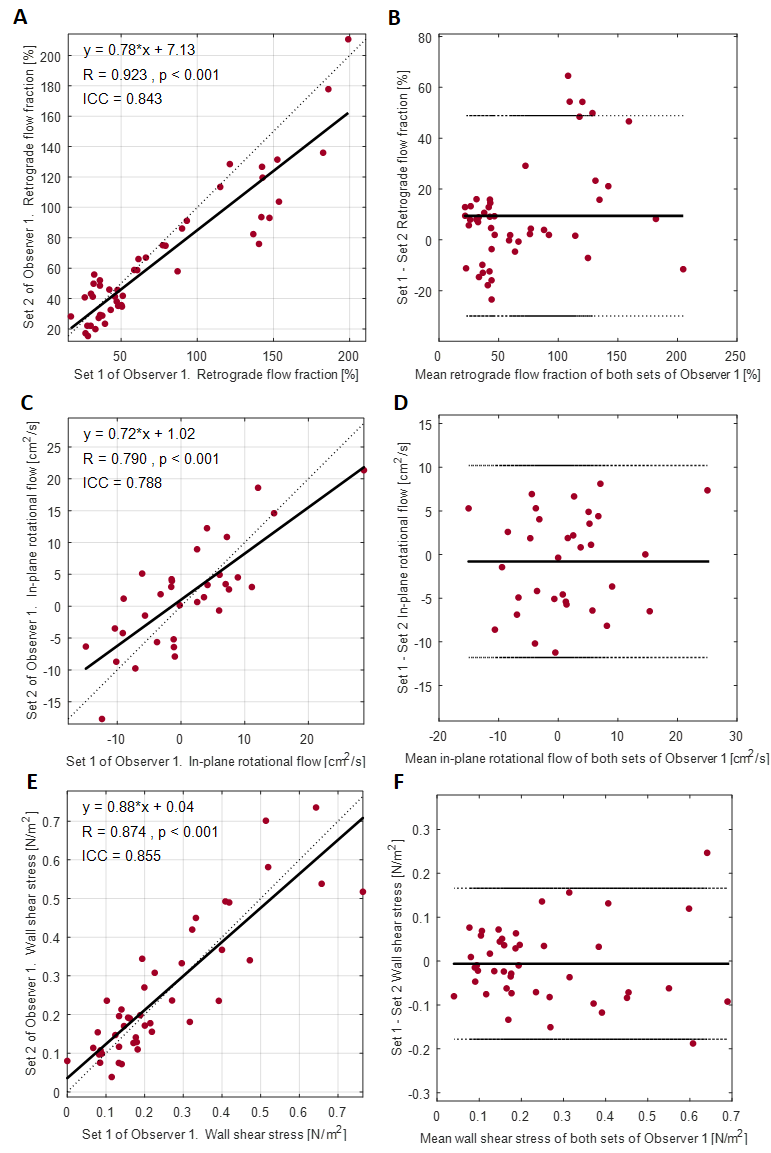** |
| Figure S6. **I*ntra-observer variability of retrograde flow fraction, in-plane rotational flow and wall shear stress.*** *Correlation (left) and Bland-Altman (right) plots for the intra-observer reproducibility of retrograde flow fraction (A and B), in-plane rotational flow (C and D) and wall shear stress (E and F). Dotted lines represent bisectors (left) and 95% confidence interval (right) while continuous lines represent best linear fit (left) and mean difference (right).* |

| **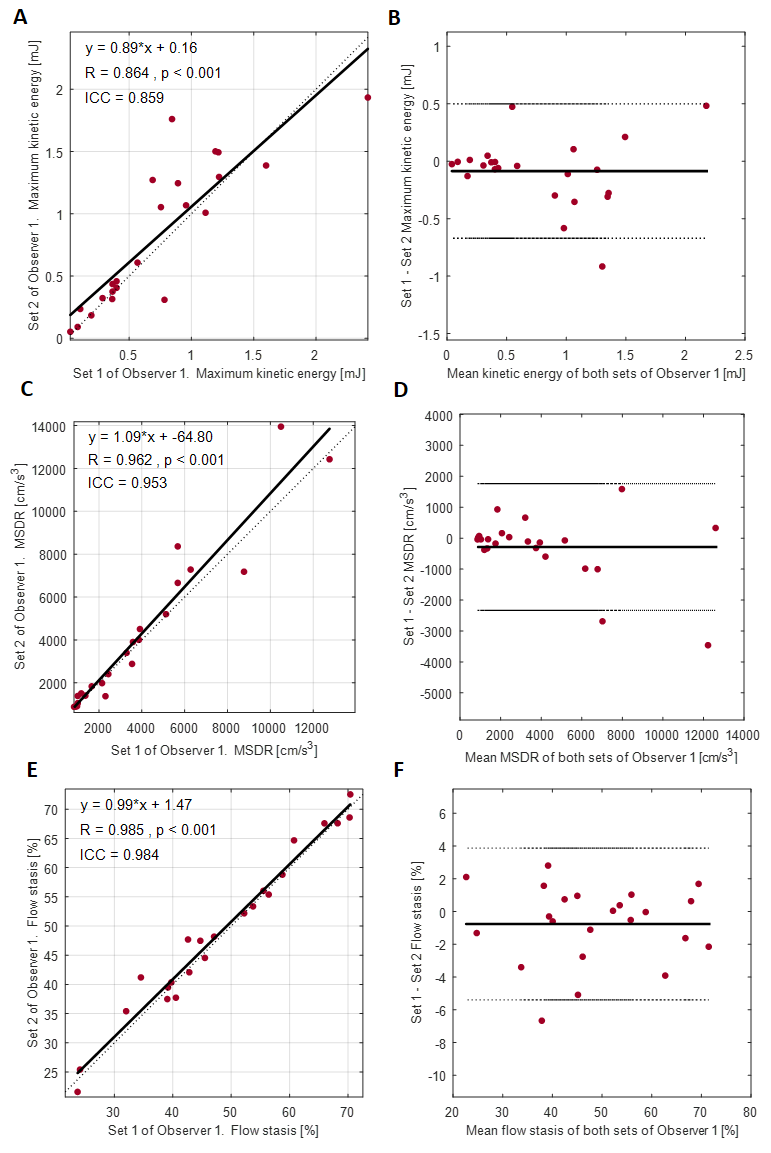** |
| --- |
| Figure S7. **I*ntra-observer variability of kinetic energy, maximum systolic deceleration rate (MSDR) and flow stasis.*** *Correlation (left) and Bland-Altman (right) plots for the intra-observer reproducibility of kinetic energy (A and B), MSDR (C and D) and flow stasis (E and F). Dotted lines represent bisectors (left) and 95% confidence interval (right) while continuous lines represent best linear fit (left) and mean difference (right).* |

**REFERENCES**

1. Rodriguez-Palomares J, Dux-Santoy L, Guala A, Kale R, Maldonado G, Teixidó-Turà G, Galian L, Huguet M, Valente F, Gutiérrez L et al. Aortic flow patterns and wall shear stress maps by 4D-flow cardiovascular magnetic resonance in the assessment of aortic dilatation in bicuspid aortic valve disease. J Cardiovasc Magn Reson. 2018;20:28. doi: 10.1186/s12968-018-0451-1.

2. Stalder AF, Russe MF, Frydrychowicz A, Bock J, Hennig J, Markl M. Quantitative 2D and 3D phase contrast MRI: Optimized analysis of blood flow and vessel wall parameters. Magn Reson Med. 2008;60:1218–1231. doi:10.1002/mrm.21778.

3. Ruiz-Munoz A, Guala A, Teixido-Tura G, Dux-Santoy L, Sao-Aviles A, Lopez-Sainz A, Chiara G, Servato L, Casas G, Gonzalez-Alujas T et al. Aortic dilatation in patients with chronic descending aorta dissection is related to maximum false-lumen systolic flow deceleration rate as evaluated by 4D-flow MRI. Eur Hear J - Cardiovasc Imaging. 2020;21. doi: 10.1093/ehjci/jez319.1018.

4. Ziegler M, Welander M, Lantz J, Lindenberger M, Bjarnegård N, Karlsson M, Ebbers T, Länne T, Dyverfeldt P. Visualizing and quantifying flow stasis in abdominal aortic aneurysms in men using 4D flow MRI. Magn Reson Imaging. 2019;57:103–110. doi:10.1016/j.mri.2018.11.003.

5. Bargiotas I, Mousseaux E, Yu WC, Venkatesh BA, Bollache E, De Cesare A, Lima JAC, Redheuil A, Kachenoura N. Estimation of aortic pulse wave transit time in cardiovascular magnetic resonance using complex wavelet cross-spectrum analysis. J Cardiovasc Magn Reson. 2015;17:65. doi:10.1186/s12968-015-0164-7.

6. Guala A, Rodriguez-Palomares J, Dux-Santoy L, Teixidó-Turà G, Maldonado G, Galian L, Huguet M, Valente F, Gutiérrez L, González-Alujas MT et al. Influence of Aortic Dilation on the Regional Aortic Stiffness of Bicuspid Aortic Valve Assessed by 4-Dimensional Flow Cardiac Magnetic Resonance: Comparison With Marfan Syndrome and Degenerative Aortic Aneurysm. JACC Cardiovasc Imaging. 2019;12:1020–1029. doi:10.1016/j.jcmg.2018.03.017.
